# Phenotypes of *Campylobacter jejuni luxS* Mutants Are Depending on Strain Background, Kind of Mutation and Experimental Conditions

**DOI:** 10.1371/journal.pone.0104399

**Published:** 2014-08-05

**Authors:** Linda Adler, Thomas Alter, Soroush Sharbati, Greta Gölz

**Affiliations:** 1 Institute of Food Hygiene, Freie Universität Berlin, Berlin, Germany; 2 Institute of Veterinary Biochemistry, Freie Universität Berlin, Berlin, Germany; Charité-University Medicine Berlin, Germany

## Abstract

Since the discovery that *Campylobacter* (*C.*) *jejuni* produces Autoinducer 2 (AI-2), various studies have been conducted to explore the function and role of AI-2 in *C. jejuni*. However, the interpretation of these analyses has been complicated by differences in strain backgrounds, kind of mutation and culture conditions used. Furthermore, all research on AI-2 dependent phenotypes has been conducted with AI-2 synthase (*luxS*) mutants. This mutation also leads to a disruption of the activated-methyl-cycle. Most studies lack sufficient complementation resulting in not knowing whether phenotypes of *luxS* mutants depend on disrupted metabolism or lack of AI-2. Additionally, no AI-2 receptor has been found yet. All this contributes to an intensive discussion about the exact role of AI-2 in *C. jejuni*. Therefore, we examined the impact of different experiment settings on three different *C. jejuni luxS* mutants on growth and motility (37°C and 42°C). Our study showed that differing phenotypes of *C. jejuni luxS* mutants depend on strain background, mutation strategy and culture conditions. Furthermore, we complemented experiments with synthetic AI-2 or homocysteine as well as the combination of both. Complementation with AI-2 and AI-2+homocysteine significantly increased the cell number of *C. jejuni* NCTC 11168Δ*luxS* in stationary phase compared to the non-complemented *C. jejuni* NCTC 11168Δ*luxS* mutant. Genetic complementation of both *C. jejuni* 81-176 *luxS* mutants resulted in wild type comparable growth curves. Also swarming ability could be partially complemented. While genetic complementation restored swarming abilities of *C. jejuni* 81-176Δ*luxS*, it did not fully restore the phenotype of *C. jejuni* 81-176::*luxS*, which indicates that compensatory mutations in other parts of the chromosome and/or potential polar effects may appear in this mutant strain. Also with neither synthetic complementation, the phenotype of the wild type-strains was achieved, suggesting yet another reason for differing phenotypes other than communication and methionine metabolism for *C. jejuni luxS* mutants.

## Introduction

Numerous bacteria communicate via the small interspecies-specific signalling molecule autoinducer-2 (AI-2) generated via LuxS [1]. This process is commonly known as Quorum sensing (QS). QS is a regulatory mechanism of gene expression, which enables bacteria to change their behaviour when the population reaches a particular cell-density. QS allows bacteria to communicate with each other and therefore coordinate their activities at a multicellular level. QS regulated processes are for example secretion of virulence factors, biofilm formation, motility and bioluminescence [2]–[4].

AI-2 is generated as a by-product via LuxS during the activated methyl cycle (AMC) [5], [6]. The AMC is an important metabolic pathway in cells. The starting compound is S-adenosyl-methionine (SAM), which is the general methyl donor. It donates its methyl group to diverse cellular components such as DNA, RNA and proteins. SAM is thereby converted to S-adenosyl-homocysteine (SAH), which is a toxic compound and has to be recycled. For recycling of SAH, two different pathways are known so far: a one-step and a two-step pathway. Only in the two-step pathway AI-2 is produced. In the two-step pathway Pfs (5′methylthioadenosine/S-adenosyl-homocysteine nucleosidase) hydrolyzes SAH to S-ribosylhomoscysteine (SRH) and adenine. LuxS catalyzes the cleavage of SRH to 4,5-dihydroxyl-2,3-pentanedion (DPD) and homocysteine [7], [8]. DPD is spontaneously cyclized into AI-2, while homocysteine is converted by MetE or MetH to methionine. Methionine is then converted by MetK into SAM [9].

In *V. harveyi*, AI-2 binds to the periplasmic binding protein LuxP. In many other bacteria e.g. *Salmonella* and *Escherichia coli*, AI-2 binds to LsrB, the ligand binding protein of an ABC transporter. So far, no homologues of the known AI-2 receptors like LuxP or LsrB were identified in *Campylobacter* spp. [10], [11].

Recently, Rader et al. [12] described that the chemoreceptor TlpB functiones as AI-2 receptor in *Helicobacter pylori*. Despite of the existence of chemoreceptors in *C. jejuni*, which would suggest the existence of a corresponding receptor, no TlpB receptor homolog has been found yet.

The existence of LuxS, as well as the LuxS-dependent AI-2 production in *C. jejuni* NCTC 11168, was first described by Elvers and Park [13]. The fact that AI-2 is a by-product of the AMC and that a receptor is yet to be found, leads to the question, if AI-2 in *C. jejuni* is indeed a true QS signal molecule. The disruption of *luxS* could lead to changing phenotypes due to the absence of AI-2 or disrupted methionine cycle. Thus, experimental analysis with *luxS* mutants needs to be complemented with AI-2 and/or a metabolic replacement substance like homocysteine (HC). Several studies of *C. jejuni luxS* mutants showed various results with diverse phenotypes in *luxS* mutants. For instance, motility and growth seems to be influenced through *luxS* disruption but in slightly different ways depending on the study design and conditions [13]–[15].

These sometimes opposing phenotypes might be due to different culture conditions. Furthermore, the authors conducted their studies with *luxS* mutants of different *C. jejuni* strains and used different mutation strategies. Additionally, most studies lack proof of complementing the *luxS* mutant strains with AI-2 and/or a metabolic substance to confirm whether resulting phenotypes are due to metabolic function of LuxS or a consequence of disrupting cell communication.

Therefore, we examined the impact of strain background, mutation strategy and culture condition on three different *C. jejuni luxS* mutants on growth and motility. Furthermore complementation experiments with synthetic AI-2 and/or homocysteine (HC) were conducted.

## Materials and Methods

### Bacterial strains and growth conditions

*Campylobacter* (*C*.) strains described in Table 1 were cultured at 37°C or 42°C in Brucella broth (BB) (BD, Heidelberg, Germany), cation adjusted Mueller-Hinton-Broth (MH) (BD) or on Mueller-Hinton blood agar plates (MHB) (Oxoid, Wesel, Germany) under microaerobic conditions (5% O_2_, 10% CO_2_) generated by an Anoxomat (Omni Life Science, Bremen, Germany). *V. harveyi* was cultured in Autoinducer bioassay medium (AB). AB medium contained 0.3M NaCl, 0.05M MgSO4, and 0.2% vitamin-free casamino-acids (Difco, BD, Heidelberg, Germany). After adjusting the pH to 7.5 with KOH the medium was sterilized by autoclaving and then allowed to cool to room temperature. Finally, 1 ml of sterile 1M potassium phosphate (pH 7.0), 1 ml of 0.1M L-arginine (free-base) and 2 ml of 50% glycerol were added per 100 ml of AB medium.

**Table 1:** Bacterial strains used in this study.

Strains	*luxS*	Description	Source or reference
*C. jejuni* 81-176	+	Wild type, virulent clinical isolate from a gastroenteritis outbreak	ATCC
*C. jejuni* NCTC 11168	+	Wild type, isolated from clinical sample in the UK in 1977	NCTC
*C. jejuni* 81-176Δ*luxS*	−	*luxS*- deletion mutant, Cm^r^	He et al. [14]
*C. jejuni* 81-176Δ*luxS* +pBQ117	+	*luxS*- deletion mutant complemented with pBQ117, Cm^r^, Km^r,^	This study
*C. jejuni* 81-176*luxS* +pBQ1015	−	*luxS*- deletion mutant with *Campylobacter* vector pBQ1015, Cm^r^, Km^r^	This study
*C. jejuni* 81-176::*luxS*	−	*luxS*- insertion mutant, Cm^r^	Quinones et al. [15]
*C. jejuni* 81-176::*luxS* +pBQ117	+	*luxS*- insertion mutant complemented with pBQ117, Cm^r^, Km^r,^	Quinones et al. [15]
*C. jejuni* 81-176::*luxS* +pBQ1015	−	*luxS*- insertion mutant with *Campylobacter* vector pBQ1015, Cm^r^, Km^r^	Quinones et al. [15]
*C. jejuni* NCTC 11168Δ*luxS*	−	*luxS*- deletion mutant, Km^r^	Corcionivoschi et al. [34]
*V. harveyi* BB152		AI-2 positive control in luminescence Bioassay	Bassler et al. [16]
*V. harveyi* BB170		AI-2 reporter in luminescence Bioassay	Bassler et al. [16]

Cm, chloramphenicol; Km, kanamycin.

The mutation of *luxS* was genetically confirmed. Therefore all *luxS* mutants were verified by PCR, followed by DNA sequencing of the amplified products. Additionally, the absence of AI-2 activity of all *luxS* mutants, as well as the presence of AI-2 in genetically complemented mutants was routinely tested in *V. harveyi* bioluminescence assay [16].

### *V. harveyi* bioluminescence assay

Overnight cultures of *C. jejuni and V. harveyi* BB152 (positive control) were diluted in BB or AB to a cell density of 1×10^8^ CFU/ml. Culture supernatants were collected and centrifuged at 8000× g for 10 min. The supernatants were sterilized by passing through a 22 µm filter (VWR, Darmstadt, Germany) and stored at −20°C until used. In parallel the absorbance was measured at the same time point to determine cell growth.

The *V. harveyi* autoinducer assay was performed as described previously [16]. The reporter strain (BB170) was grown over night in AB medium and diluted (1∶5000) into fresh AB medium. CFS and uninoculated AB respectively BB medium were then added to the diluted *V. harveyi* culture at 10% (v/v) final concentration. As another positive control AI-2 (10 µM) alone was tested. The reporter strain with CFS, AI-2 or uninoculated media were incubated at 30°C with aeration (750 rpm). After 4 hours of incubation, luminescence of 100 µl aliquots in microtiter plates were measured (10 s per well) using Luminometer (CentroPro, Berthold, Bad Wildbach). For each of three experiments, triplicates of relative light units (RLU) were measured. n- fold luminescence induction values were calculated from RLU obtained with conditioned CFS vs. RLU obtained with sterile medium.

### Chemical complementation

AI-2 activity was quantified with the bioluminescence assay and compared to wild-type *C. jejuni* grown to an OD600 nm of 1.0, at which maximal AI-2 activity was obtained. To test for complementation of growth and motility, AI-2 (OMM Scientific, Dallas, USA) at a physiological concentration of 10 µM and non-limiting concentration of 100 µM was used. Homocysteine (HC; Sigma Aldrich, St. Louis, USA) was tested at 1 µM, 10 µM and 100 µM.

To exclude non-specific effects of AI-2 and homocysteine, the same concentrations were added to wt strains.

### Genetic complementation

To exclude potential polar effects in the mutant strains, genetic complementation was performed exemplarily in the insertion and deletion mutant of strain *C. jejuni* 81-176. The complemented *C. jejuni* 81-176::*luxS* strain was kindly provided by Quiñones et al. [15].

Quiñones et al. [15] complemented the *luxS* mutation in strain *C. jejuni* 81-176::*luxS* with pBQ117 (plasmid pWM1015 containing a 1.3-kb fragment with the promoter-proximal region and intact *luxS* gene from strain 81-176).

In this study both plasmids, pWM1015 and pBQ117, were introduced into *C. jejuni* 81-176Δ*luxS* by electroporation [17].

### Growth assay

For growth assays, cultures of wild type (wt) and *luxS* mutants of *C. jejuni* NCTC 11168 and *C. jejuni* 81-176 strains were grown overnight in BB or MH at 37°C. Precultures were inoculated in BB or MH to approx. 2×10^5^ CFU/ml and incubated under microaerobic conditions at 37°C and 42°C. For chemical complementation assays 10 µM AI-2, HC or AI-2+HC were added to the cultures.

Numbers of viable bacteria were determined over 48 h by plating serial dilutions of the bacterial suspensions. Results reported are the average of at least three independent assays.

### Swarming assay

For swarming either BB containing 0.4% agar (BBA) or MH containing 0.4% agar (MHA) were used. The swarming ability of *C. jejuni luxS* mutants was investigated at 37°C and 42°C on swarming plates. For chemical complementation 10 µM each of AI-2, HC or AI-2+HC were added to the molten agar. Overnight cultures of *C. jejuni* strains were adjusted to 10^8^ CFU/ml and 1 µl dropped on BBA or MHA. After 24 h incubation at 37°C or 42°C the diameters of the swarming halos were measured. Halos of *luxS* mutants were normalized to the wild type halos (100%). Results reported are the median of six independent assays.

### Statistical analysis

For statistical analyses, all experiments were repeated at least three times in three independent experiments.

Statistical analyses were performed using GraphPad Prism v6.0 (GraphPad Prism, San Diego, USA). To calculate significant differences a two- tailed Mann-Whitney test was used. For all statistical analyses, a confidence level of 95% was defined.

## Results

### Growth assays

#### Strain background

**Figure 1:**
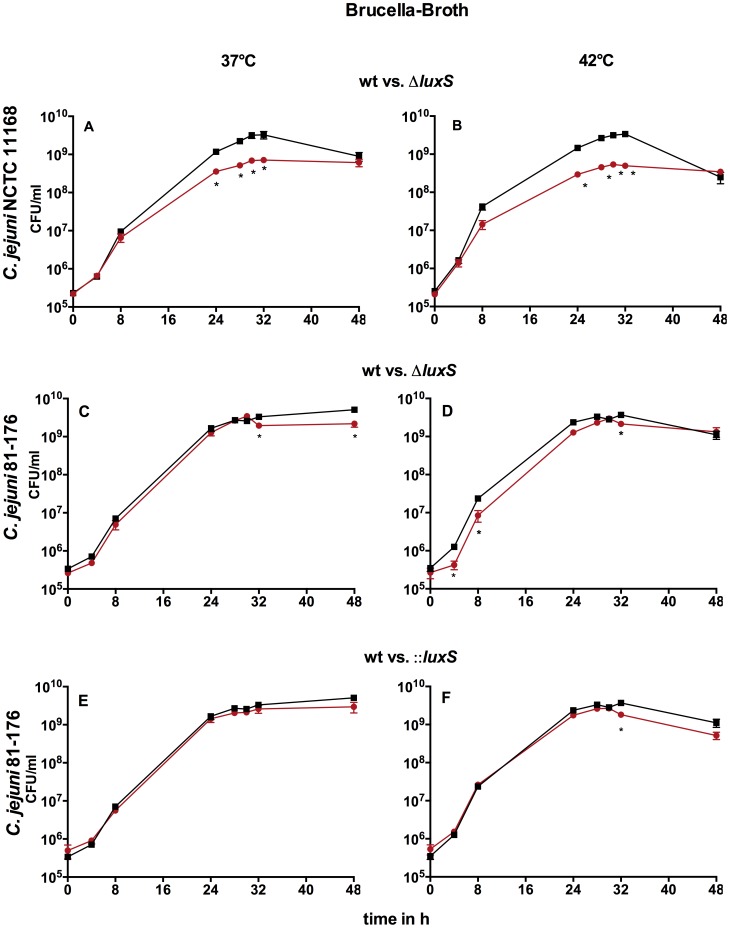
Growth of *C. jejuni* NCTC 11168 and *C. jejuni* 81-176 wt and *luxS* mutants at 37°C and 42°C in BB: A–B *C. jejuni* NCTC 11168 wt/Δ*luxS*, C–D *C. jejuni* 81-176 wt/Δ*luxS*, E–F *C. jejuni* 81-176 wt/::*luxS*; black- wild type, red- *luxS* mutant; shown are the means ± SD (n = 3), _*_ -p<0.05 (Mann-Whitney-U test).

Growth of both *C. jejuni* wild type strains did not differ at 37°C and 42°C, whereas growth profiles of the three *luxS* mutants were not equal (Fig. 1A–F). Compared to the wild type the Δ*luxS* mutant of *C. jejuni* NCTC 11168 showed significantly reduced cell numbers within mid-exponential (8 h) and mid-stationary phase (32 h) while cell numbers converged during late stationary phase (48 h) at both temperatures (Fig. 1A–B). *C. jejuni* 81-176Δ*luxS* and::*luxS* mutants showed comparable cell numbers to the wild type at 37°C with a slight decrease at late stationary phase (Fig. 1C/E). At 42°C *C. jejuni* 81-176Δ*luxS* showed significantly decreased cell numbers in exponential and stationary phase, but *C. jejuni* NCTC 11168Δ*luxS* showed much lower levels (Fig. 1D). However, the cell number of *C. jejuni* 81-176::*luxS* only decreased significantly at mid-stationary phase at 42°C (Fig. 1F). These results demonstrate that strain background has an impact on growth profiles of *C. jejuni luxS* mutants.

#### Culture conditions

**Figure 2:**
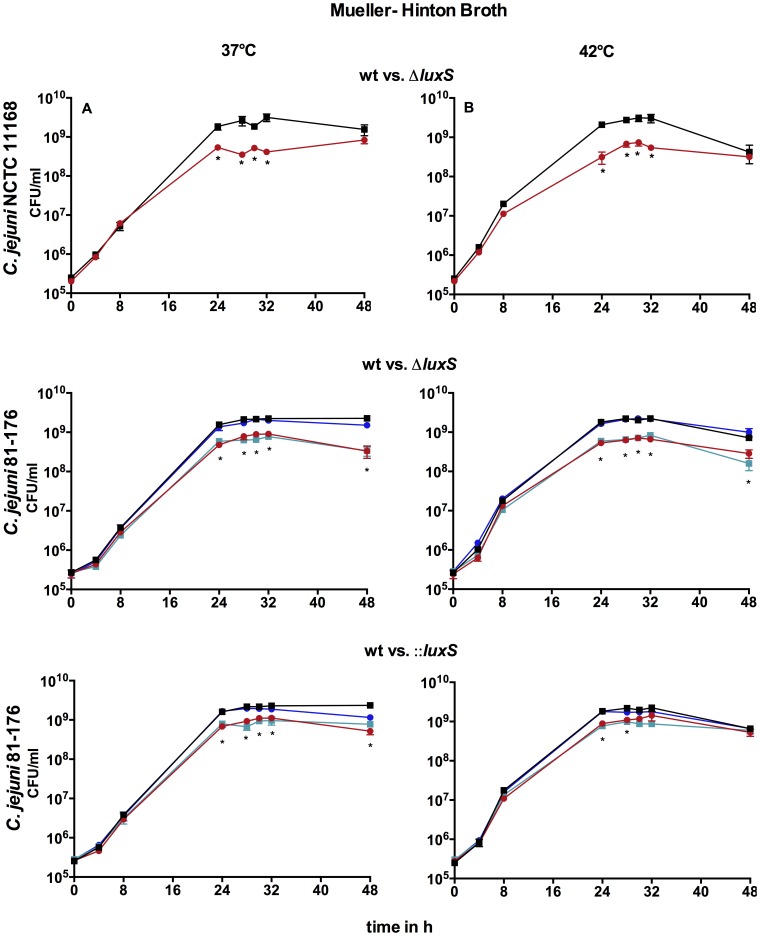
Growth of *C. jejuni* NCTC 11168 and *C. jejuni* 81-176 wt, *luxS* mutants and genetic complemented *C. jejuni* 81-176 mutants at 37°C and 42°C in MH: A–B *C. jejuni* NCTC 11168 wt/Δ*luxS*, C–D *C. jejuni* 81-176 wt/Δ*luxS*, E–F *C. jejuni* 81176 wt/::*luxS*; black- wild type, red- *luxS* mutant, blue- *luxS*+ pBQ117, turquoise- *luxS*+ pBQ1015; shown are the means ± SD (n = 3), _*_ -p<0.05 (Mann-Whitney-U test).

When comparing growth of *C. jejuni* NCTC 11168 wt and mutant in MH versus BB it becomes obvious that the growth profiles were quite similar between these media at both temperatures (Fig. 1A–B and 2A–B). In contrast, growth of *C. jejuni* 81-176 wt was slightly reduced in late stationary phase in MH. Both mutants of strain *C. jejuni* 81-176 showed growth defects in MH at 37°C that were not exhibited in BB (Fig. 2C–F). In addition, growth profiles of *C. jejuni* 81-176Δ*luxS* mutants differed between MH and BB, thus the cell numbers of *C. jejuni* 81-176Δ*luxS* in MH were substantially reduced in the stationary phase while cell numbers in BB showed only a partially slight reduction. This indicates that composition of media could influences growth of *luxS* mutants. Temperature influences growth profiles of the *C. jejuni* 81-176::*luxS* mutant strain in MH. However, temperature did not have an impact on growth of the other *C. jejuni luxS* mutants.

#### Impact of mutation strategy on growth of *luxS* mutants

To investigate the influence of mutation strategy, three different *C. jejuni luxS* mutants were used. Considering growth profiles of *C. jejuni* in MH the influences of mutation strategy became apparent. The Δ*luxS* mutants of both *C. jejuni* NCTC 11168 and *C. jejuni* 81-176 showed growth defects which were not exhibited in the insertional mutant *C. jejuni* 81-176::*luxS* at 42°C (Fig. 2D/F). These results demonstrated the impact of mutation strategy.

#### Genetic complementation

Based on the varying growth profiles between *C. jejuni* 81-176 wt and its *luxS* mutant strains in MH, genetic complementation of the *C. jejuni* 81-176 mutant strains were conducted. Genetic complementation of both *C. jejuni* 81-176 *luxS* mutants in MH resulted in wild type comparable growth curves (Fig. 2). Introduction of the isogenic plasmid pWM1015 alone did not alter growth of *C. jejuni* 81-176 mutants. This indicates that no polar effects caused varying growth profiles of *C. jejuni* 81-176 *luxS* mutants in MH.

#### Chemical complementation

**Figure 3:**
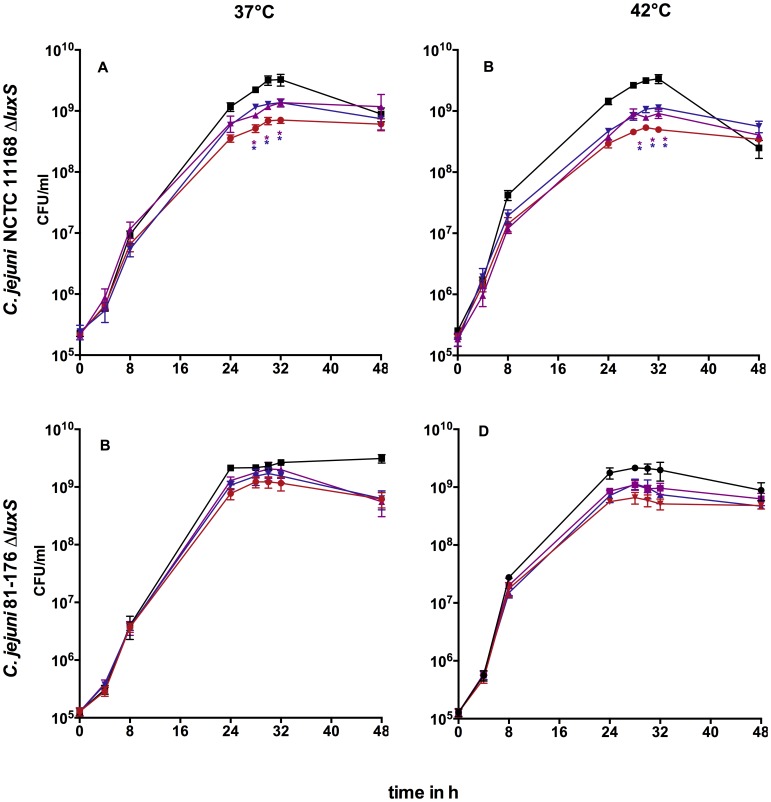
Growth curves of chemically complemented *C. jejuni* in BB (*C. jejuni* NCTC 11168Δ*luxS*) or MH (*C. jejuni* 81-176Δ*luxS*): A/C 37°C, B/D- 42°C; black- wild type, red- Δ*luxS*, purple- Δ*luxS*+AI-2, blue- Δ*luxS*+AI-2+HC; shown are mean ± SD (n = 5), _*_- p<0.05 compared to Δ*luxS* (Mann-Whitney-U test).

Because of the varying growth profiles between *C. jejuni* NCTC 11168 wt and its Δ*luxS* mutant we examined if the addition of exogenous AI-2 and HC influences growth of *C. jejuni* NCTC 11168Δ*luxS* in BB. Complementation with AI-2 (10 µM) and AI-2+HC (both 10 µM) significantly increased the cell number of *C. jejuni* NCTC 11168Δ*luxS* in stationary phase at 37°C and 42°C compared to the non-complemented Δ*luxS* mutant (Fig. 3A/B). In contrast, HC alone did not show any significant effect on cell numbers at any of the investigated temperatures (data not shown). Full restoration of wild type cell numbers was not achieved by the chemical complementation strategy used in *luxS* mutants indicating that alternative mechanisms for these different phenotypes exist. The same effects have been observed with 100 µM AI-2 and 100 µM HC (data not shown). Non-specific effects through AI-2 and homocysteine could be excluded since no alteration in phenotypes of the wild type was observed (data not shown).

With the addition of AI-2 and AI-2+HC to strain *C. jejuni* 81-176Δ*luxS* in MH media slightly increased cell numbers during stationary phase were observed at both temperatures, but these were not statistically significant (Fig. 3C/D). The addition of HC alone did not lead to increased cell numbers (data not shown).

### Swarming ability

#### Strain background

**Figure 4:**
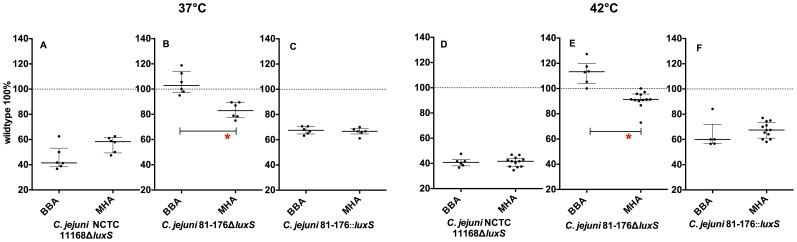
Swarming ability of *C. jejuni luxS* mutants on different media: A–C 37°C, D–F 42°C; shown are the normalized medians with interquartile range (n = 6), _*_ -p<0.05, (Mann-Whitney-U test); calculation of significance: BBA vs. MHA.

**Figure 5:**
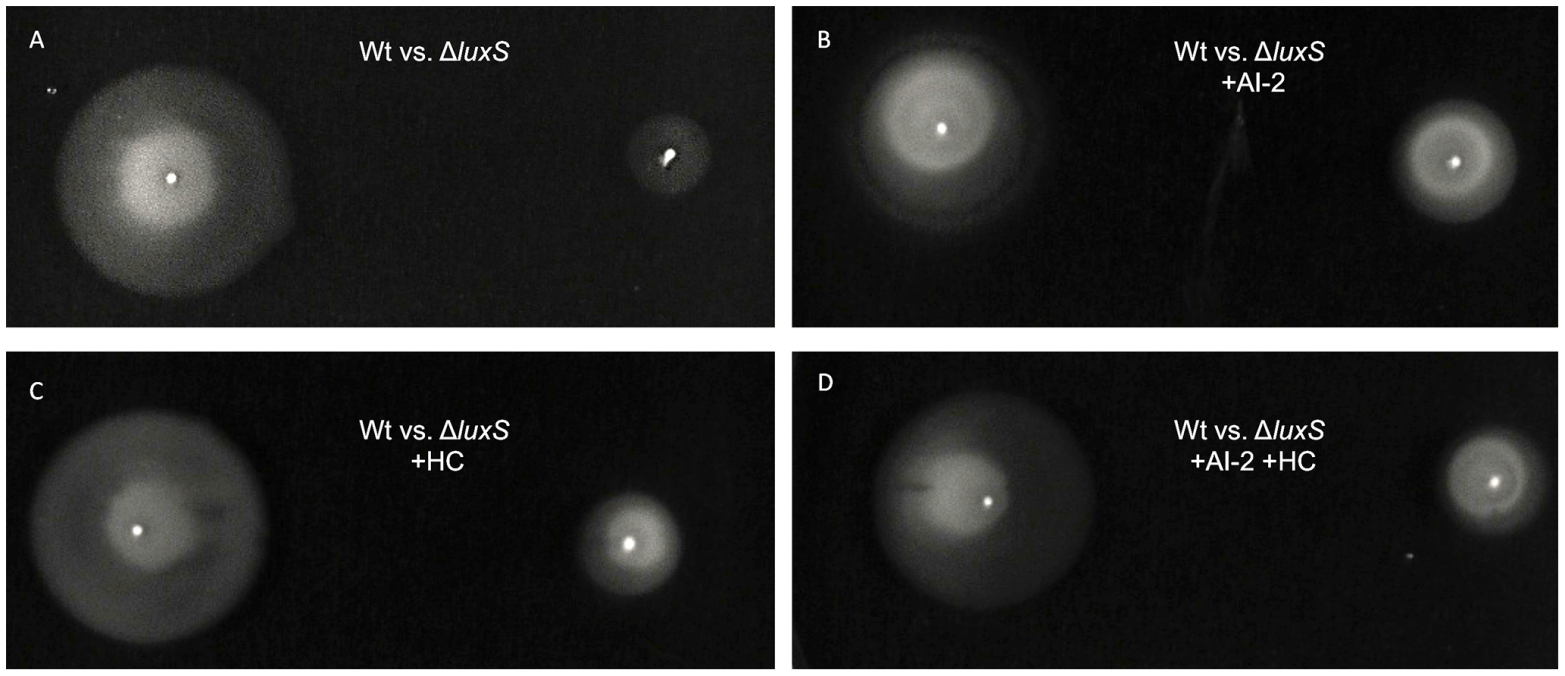
Swarming halos of *C. jejuni* 11168 wt and Δ*luxS* mutant in BBA (37°C): A- wt vs. Δ*luxS*, B- wt vs. Δ*luxS*+ AI-2, C- wt vs. Δ*luxS*+ HC and D- wt vs. Δ*luxS*+ AI-2+ HC.

Swarming ability *of C. jejuni* wild types did not differ between the different strains (data not shown). Swarming ability of *luxS* mutants were normalized to the wild type (100%). *C. jejuni* NCTC 11168Δ*luxS* showed reduced swarming ability (Fig. 4A/D, Fig. 5A) (approx. 42% of wt swarming in BBA). In contrast, *C. jejuni* 81-176Δ*luxS* showed no significant reduction in swarming ability in BBA (Fig. 4B/E). Our data clearly indicate the influences of strain background on swarming ability of *C. jejuni* Δ*luxS* mutants under these conditions (Fig. 4A–F).

#### Impact of mutation strategy on swarming ability of luxS mutants

Insertion and deletion of *luxS* in strain *C. jejuni* 81-176 resulted in different swarming abilities. In our experimental setting, only the *C. jejuni* 81-176::*luxS* mutant exhibited smaller swarming halos (Fig. 4C/F) compared to the wt but not the deletion mutant of this strain (Fig. 4B/E). Our data indicate that mutation strategy influences the ability to swarm in *C. jejuni* 81-176 *luxS* mutants.

#### Culture conditions

At 42°C diameters of swarming halos of all strains and their mutants increased compared to halos at 37°C (data not shown). Neither temperature nor media influenced the reduced swarming abilities of *C. jejuni* NCTC 11168Δ*luxS* (Fig. 4A/D) and *C. jejuni* 81-176::*luxS* (Fig. 4C/F) compared to their wt. In contrast, swarming ability of *C. jejuni* 81-176Δ*luxS* (Fig. 4B/E) was slightly increased compared to the wt in BBA at both temperatures and decreased in MHA. The difference in swarming halos of *C. jejuni* 81-176Δ*luxS* was statistically significant between both media. This result indicates that culture conditions could have an impact on swarming ability of *C. jejuni luxS* mutants.

#### Chemical complementation in BB

**Figure 6:**
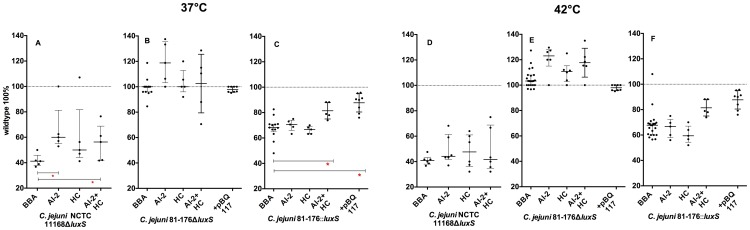
Swarming ability of complemented *C. jejuni luxS* mutants on BBA: A–C 37°C, D–F 42°C, complementation with: AI-2, HC, AI-2+HC and pBQ117; shown are the normalized median and interquartile range (n = 6), _*_ -p<0.05 (Mann-Whitney-U test).

Hence, we examined if the addition of exogenous AI-2 and HC influences the reduced swarming of *C. jejuni luxS* mutants on BBA at 37°C and 42°C (Fig. 6). In BBA complementation with AI-2 and AI-2+HC contributed to an increased swarming ability compared to the non-complemented mutant of *C. jejuni* NCTC 11168Δ*luxS* at 37°C (Fig. 6) but not at 42°C (Fig. 6A/D). However, the addition of HC alone did not alter the swarming ability at both temperatures. Only the addition of both AI-2+HC to *C. jejuni* 81-176::*luxS* mutant increased the swarming motility at 37°C (Fig. 6C), while the swarming ability of *C. jejuni* 81-176Δ*luxS* was not significantly altered by any condition investigated (Fig. 6B/F). Our data implicate that partial complementation of *luxS* mutants is possible in BBA depending on temperature and strain background. Complementation only occurs if AI-2 is admitted. With neither chemical complementation of *luxS* mutants, the phenotype of the wild type-strains was achieved.

#### Chemically complementation in MH

**Figure 7:**
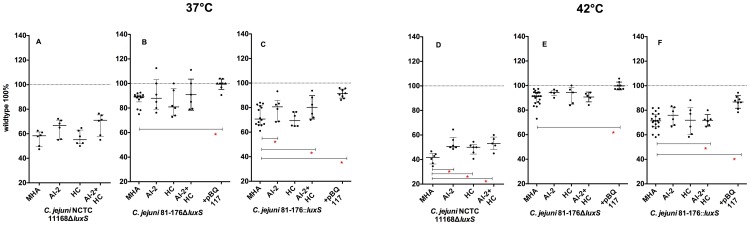
Swarming ability of complemented *C. jejuni luxS* mutants on MHA: A–C 37°C, C–F 42°C, complementation with: AI-2, HC, AI-2+HC and pBQ117 shown are the normalized medians and interquartile range (n = 6), _*_ -p<0.05 (Mann-Whitney-U test).

The swarming ability of *C. jejuni* NCTC 11168Δ*luxS* was increased through the addition of AI-2, HC and AI-2+HC at 42°C compared to the non-complemented mutant strain on MHA (Fig. 7). At 37°C only a slightly increased swarming ability could be observed through the addition of AI-2 and AI-2+HC. However, the addition of AI-2 to *C. jejuni* 81-176::*luxS* yielded increased swarming motility at both temperatures. Furthermore, with the addition of AI-2+HC swarming motility could also be increased at 37°C in this mutant. In contrast, the swarming ability of *C. jejuni* 81-176Δ*luxS* was not significantly changed by any complementation investigated. With neither chemical complementation, the phenotype of the wild type-strains was achieved. Complementation in MHA is likely to occur but depends on strain background and mutation strategy.

#### Genetic complementation

Introduction of the isogenic plasmid pWM1015 alone did not alter swarming ability of *C. jejuni* 81-176 mutants. Genetic complementation (+pBQ117) restored swarming abilities of *C. jejuni* 81-176Δ*luxS* (Fig. 7B/D) in MH, whereas in strain *C. jejuni* 81-176::*luxS* genetic complementation did not fully restore the phenotype at 37°C and 42°C in both media (Fig. 6A/D and Fig. 7A/D). The incomplete restoration hints at appearing polar effects in this mutant strain.

## Discussion

Since the discovery that *C. jejuni* produces AI-2, various studies have been conducted to explore the function and role of AI-2 in *C. jejuni* [18], [19]. However, the interpretation of these analyses has been complicated by differences in strain background, kind of mutation and culture conditions. Furthermore most studies lack sufficient complementation resulting in not knowing whether phenotypes of *luxS* mutants depend on disrupted metabolism or lack of AI-2. Additionally, no AI-2 receptor has been found yet. All this contributes to an intensive discussion about the exact role of AI-2 in *C. jejuni*.

In the literature, various motility and growth phenotypes have been described for *C. jejuni luxS* mutants [14], [15], [20]. Therefore, we investigated if the strain background, kind of mutation and different culture conditions impact the occurring phenotypes in *C. jejuni luxS* mutants.

To verify that our *luxS*-mutant strain truly deficient in AI-2 production a *V. harveyi* bioluminescence reporter assay was conducted. *V. harveyi* only responds to the borate diester derived from (2S,4S)-THMF [9]. The wild type strain of *C. jejuni* as well as the synthetic AI-2 exhibit positive signals in this reporter assay, indicating that the absence of a positive signal (in the reporter assay) of the mutant strain is equatable to the absence of AI-2 production. Further the *V. harveyi* reporter assay indicates that the synthetic AI-2 contains a similar equilibrium of AI-2 as the one produced by *C. jejuni* NCTC 11168.

### Culture conditions

During growth of *C. jejuni* wild types there were no significant differences observed in BB and MH. Also Ng et al. [21] described that there was no significant difference between cell numbers of *C. jejuni* among these basal media. Growth and swarming abilities of *C. jejuni* NCTC 11168Δ*luxS* were quite similar in BB and MH at both temperatures (37°C and 42°C), which leads to the assumption that these culture conditions do not have an impact on the *luxS* mutant phenotype of this strain.

Further, He et al. [14] described that the cell numbers of *C. jejuni* 81-176 differed between wild type and Δ*luxS* mutant at 37°C and 42°C in the mid-exponential phase, while cell numbers converged in late stationary-phase in MH medium. In our study, we also observed reduced cell numbers of *C. jejuni* 81-176Δ*luxS* in exponential as well as in late stationary phase in MH at both temperatures. In contrast, growth of *C. jejuni* 81-176Δ*luxS* in BB only showed significant differences to growth of wild type in late stationary phase at 37°C. These results demonstrate that the choice of the culture medium has a large impact on the resulting phenotypes of *C. jejuni* 81-176 *luxS* mutants. Growth of the *C. jejuni* 81-176::*luxS* is also influenced by temperature in MH. However, growth curves at 37°C and 42°C are quite equal in *C. jejuni* 11168Δ*luxS* and *C. jejuni* 81-176Δ*luxS*, which indicate that growth differences are independent of temperature but dependent on culture media for deletion mutant strains. Motility observation confirmed this assumption as well. Like He et al. [14] we observed reduced swarming abilities of *C. jejuni* 81-176Δ*luxS* at 37°C on MHA media in contrast to the wild type. However, this phenotype was only observed on MHA media but not on BBA. The components of these two different basal mediums differ. For example only BB contains dextrose. Wang et al. [22] showed that glucose affected the gene expression of *luxS* in *E. coli*. Furthermore, they observed that the expression of *pfs* was reduced by the presence of glucose. Our findings clearly illustrate that resulting phenotypes of *C. jejuni luxS* mutants can be influenced by the choice of culture medium and components in media could also influence resulting phenotypes of *C. jejuni luxS* mutants.

### Strain background

*C. jejuni* is a highly diverse species [23]. To investigate the influence of strain background on phenotypes of *luxS* mutants we used *C. jejuni* NCTC 11168 and *C. jejuni* 81-176Δ*luxS* mutants. Comparing these phenotypes we observed differences between *luxS* mutants of *C. jejuni* NCTC 11168 and *C. jejuni* 81-176 especially in BB (Fig. 1A–D). Also, the comparison of *luxS* mutant strains of *C. jejuni* 81-176 used by He et al. [14] and *C. jejuni* NCTC 11168 used by Elvers and Park [13] revealed different outcomes in terms of growth. Comparing wild types of *C. jejuni* in our study, cell numbers of strain *C. jejuni* NCTC 11168 decline under cell numbers of strain *C. jejuni* 81-176 in late stationary phase. Cell numbers in all other time points did not differ between these two wild type strains, whereas cell numbers of *C. jejuni* NCTC 11168Δ*luxS* in early stationary phase in BB are lower than cell numbers of *C. jejuni* 81-176Δ*luxS*. Furthermore, the ability to swarm differed between the *luxS* mutants of both strains. Here, we observed that *C. jejuni* NCTC 11168Δ*luxS* exhibits the greatest reduction of swarming ability, whereas *C. jejuni* 81-176Δ*luxS* did not exhibit reduced swarming abilities at all in BB. The different observed phenotypes in *C. jejuni* NCTC 11168 and *C. jejuni* 81-176 may be a consequence of genetic diversity between these strains [24]. For instance previous analysis of the complete flagellin glycosylation locus of *C. jejuni* strain 81-176 revealed a less complex genomic organization than the corresponding region in the genome of strain *C. jejuni* NCTC 11168 [25]. In addition, Dugar et al. [17] identified strain-specific transcriptome organization and sRNAs that could contribute to differential gene regulation among these strains.

### Mutation strategy

**Figure 8:**
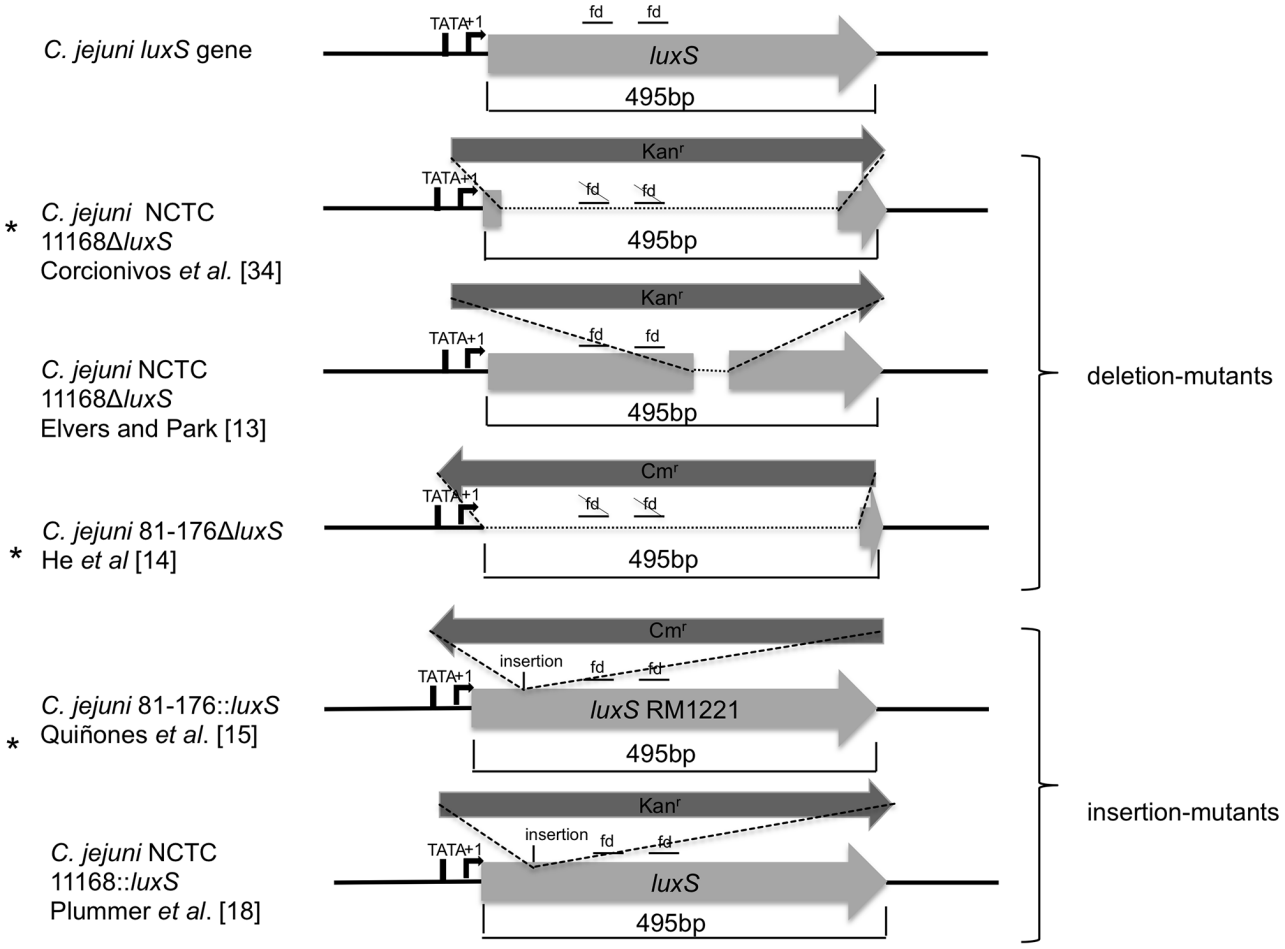
Comparison of mutation strategy of *C. jejuni luxS* mutants.

An explanation for the differences in growth profiles between *C. jejuni* NCTC 11168 and *C. jejuni* 81-176 *luxS* mutants might be the genetic differences between these two strains. However, it seems equally probable that the phenotypic differences observed were due to different mutation strategies applied. Previously Haigh *et al.* [26] showed that mutation design and strain background influenced phenotype *of E. coli luxS* mutants. The authors concluded that one explanation could be the different orientation of antibiotic resistance cassette in the mutants. The kanamycin resistance cassette of *C. jejuni* NCTC 11168Δ*luxS* is orientated in the same direction as the *luxS* gene, whereas the *C. jejuni* 81-176Δ*luxS* mutant has the chloramphenicol resistance cassette in the opposite direction of the deleted *luxS* gene. Both mutants had reduced cell numbers when cultured in MH but only the *luxS* mutant of *C. jejuni* NCTC 11168 showed lesser swarming abilities compared to the corresponding wild type. Recently, it has been demonstrated that the regulatory small RNA MicA is located closely upstream of the *E. coli luxS* gene [27], [28] and could be an obvious target for polar effects of a *luxS* mutation. In *C. jejuni*, Dugar et al. [17] showed that a small RNA is located downstream of the *luxS* gene, but no function of this small RNA has been described so far. However, the expression of this small RNA could be influenced in a mutation strategy dependent manner. Since this molecule can have regulatory effects, its dysregulation can affect other pathways. Another reason for the observed different phenotypes could be the size of the deleted region. In contrast to our results, the *C. jejuni* NCTC 11168Δ*luxS*-mutant constructed by Elvers and Park [13] showed similar growth compared to the wt at 37°C. The Δ*luxS* mutant of Elvers and Park [13] has the same strain background and orientation of antibiotic resistance cassette as the Δ*luxS* mutant used in this study. However, the mutant used in our study has a larger deletion region, including those with functional domains from *luxS* (Fig. 8 illustrated the differences in mutation strategies of *C. jejuni luxS* mutants), whereas the mutant from Elvers and Park [13] still retains the functional domain regions. Even though the functionality relating to AI-2 production is disabled, it cannot be ruled out that other regions within this sequence exert an influence on other processes. Also the *C. jejuni* NCTC 11168::*luxS* mutant described by Plummer et al. [20] (an insertion mutant up-stream of the functional domains) showed similar growth like the wt. However, the mutant of Plummer et al. [20] and Elvers and Park [13] exhibited decreased motility. Examining the mutant in the current study, decreased motility haloes in semisolid media have been observed, which indicates that disruption of *luxS* causes a reduction of swarming ability in *C. jejuni* NCTC 11168 whether or not the functional domain regions are deleted.

To investigate the influence of mutation strategy on phenotypes of *C. jejuni luxS* mutants we additionally conducted our study with an insertion mutant of *C. jejuni* 81-176. The resistance cassette of this mutant strain is also (like in *C. jejuni* 81-176Δ*luxS*) orientated in the opposite direction of the *luxS* gene. By examining growth in MH at 37°C and 42°C cell numbers of the deletion mutant are smaller than cell numbers of insertion mutant in stationary phase. Again, one reason could be the lack of the region among the functional domains within *luxS* in the deletion mutant, whereas this region is still present in the insertion mutant of *C. jejuni* 81-176. Additionally, Quinones et al. [15] replaced the *luxS* gene of strain 81-176 by a mutated *luxS* gene of the *C. jejuni* strain RM1221, which is another possible explanation for differing phenotypes of *luxS* mutants. Furthermore, Quiñones et al. [15] used larger up- and downstream regions of *luxS* for their mutation construct. Even though there is a high DNA sequence identity (96.8%) between the *luxS* genes of strains RM1221 and 81-176, there are some differences which might influence phenotypes of *luxS* mutants.

The observation of swarming ability additionally indicates the importance of mutation strategy. Like Quiñones et al. [15] we observed a significantly reduced swarming ability of *C. jejuni* 81-176::*luxS* mutant compared to the wild type on BBA, whereas the swarming ability of *C. jejuni* 81-176Δ*luxS* is not reduced on BBA in contrast to the wt.

While genetic complementation restored swarming abilities of *C. jejuni* 81-176Δ*luxS*, genetic complementation restored swarming abilities of *C. jejuni* 81-176::*luxS* only partial. The phenotype of wt strain *C. jejuni* 81-176 was not fully achieved. The incomplete restoration was probably caused by polar effects in this insertion mutant strain. Also Haigh et al. [26] argued that insertion may always result in adverse polar effects.

Combined, these findings suggest that growth deficits of *C. jejuni luxS* mutants might be associated with deletion of a larger region of *luxS*, while motility might be influenced by polar or compensatory mutation effects of the *luxS* mutation.

### Complementation

It remains unclear whether phenotypes of *luxS* mutants are due to the lack of AI-2 or result from the metabolic deficits caused by disruption of this enzyme in the activated methyl cycle. Additionally, occurring phenotypes may be appearing through polar effects depending on the kind of mutation. Genetic complementation of both *C. jejuni* 81-176 *luxS* mutants resulted in wild type comparable growth curves, which indicates that no polar effects influence growth of *C. jejuni* 81-176 *luxS* mutants.

Therefore, we investigated the phenotypes of *C. jejuni luxS* mutants chemically complemented with AI-2, HC and AI-2+HC (Fig. 3). Growth of *luxS* mutants could be partially complemented by AI-2 but not to wt level, implicating that altered phenotypes of *luxS* mutants not solely occur as a consequence of lacking AI-2. The addition of AI-2 and AI-2+HC to mutant strain *C. jejuni* 81-176Δ*luxS* leads to slightly increased cell numbers, too. Addition of HC to the mutant strains did not alter the *luxS* mutant phenotype indicating that disruption of the activated methyl cycle downstream of LuxS might not be responsible for the observed phenotypes (data not shown). The accumulation of components upstream of LuxS within the AMC as well as other unknown functions of LuxS could also have an impact on the observed phenotypes.

Furthermore, in solution, DPD exists in an equilibrium that contains diastereomeric mixtures of dihydroxytetrahydrofurans (DHMF) and tetrahydroxytetrahydrofurans (THMF) through cyclization and hydration [29].

A peculiarity of AI-2 signaling is that diverse bacteria have different AI-2 receptors which recognize distinct forms of AI-2. For example, *V. harveyi* responds to the borate diester derived from (2S,4S)-THMF [30], [31], whereas *Salmonella* Typhimurium [9], *Sinorhizobium meliloti* [32] and *Yersinia pestis* [33] respond to (2R,4S)-THMF. Thereby it is possible that the DPD used in this study might not have harboured adequate amounts of the relevant DPD variant for *C. jejuni*. However, as the synthetic DPD also induced luminescence in the *V. harveyi* assay, it can be concluded that AI-2 produced by *C. jejuni* and synthetic DPD contained a similar variation of AI-2. The response observed following chemical complementation would suggest the existence of the adequate structure of AI-2 for receptor recognition. Nevertheless it remains unclear if the lack of complete complementation could be caused by an inappropriate chemically equilibrium of the AI-2 structures.

We observed significantly increased swarming ability at 37°C by chemical complementation with AI-2 and AI-2+HC in *C. jejuni* NCTC 11168Δ*luxS* on BBA and in *C. jejuni* 81-176::*luxS* on MHA. While genetic complementation restored swarming abilities of *C. jejuni* 81-176Δ*luxS*, it did not completely restore phenotype of *C. jejuni* 81-176::*luxS*. By the addition of AI-2, the same swarming abilitiy as shown for the genetic complemented mutant was achieved. These data indicate that reduced swarming abilities are partially due to polar mutation effects in *C. jejuni* 81-176::*luxS*, but could be partially complemented by exogenous AI-2.

## Conclusions

Our study provides a clue why literature about phenotypes of *C. jejuni luxS* mutants is extremely contradictory. Our analyses demonstrated that occurring phenotypes of *C. jejuni luxS* mutants are depending on strain background, kind of mutations and experimental conditions (Table 2).

**Table 2:** Phenotypes (growth and swarming ability) of *luxS* mutants and its chemical complementation.

*C. jejuni* strains	Kind of mutation	Medium	Temp.	Motility of *luxS* mutant vs. wt	Complemented with			Growth of *luxS* mutant vs. wt	Complemented with		
					AI-2	HC	AI-2+HC		AI-2	HC	AI-2+HC
NCTC 11168	Δ*luxS*	BB	37°C	↓	↑	=	↑	(↓)	↑	=	↑
			42°C	↓	=	=	=	(↓)	↑	=	↑
		MH	37°C	↓	=	=	=	↓	n.a	n.a	n.a
			42°C	↓	↑	↑	↑	↓	n.a	n.a	n.a
81-176	Δ*luxS*	BB	37°C	=	n.a	n.a	n.a	↓	n.a	n.a	n.a
			42°C	=	n.a	n.a	n.a	↓	n.a	n.a	n.a
		MH	37°C	↓	=	=	=	↓	↑	=	↑
			42°C	↓	=	=	=	↓	↑	=	↑
81-176	::*luxS*	BB	37°C	↓	=	=	↑	=	n.a	n.a	n.a
			42°C	↓	=	=	=	(↓)	n.a	n.a	n.a
		MH	37°C	↓	↑	=	↑	(↓)	n.a	n.a	n.a
			42°C	↓	↑	=	=	=	n.a	n.a	n.a

↑: increased, ↓:reduced, (↓): slightly reduced, = : similar, n.a: not analysed, MH: Mueller-Hinton, BB: Brucella Broth, Temp.: temperature.

Further, some *luxS* mutant phenotypes could be partially complemented by AI-2, even though not to wild type levels, suggesting that *C. jejuni* can regulate its behaviour by AI-2 dependent Quorum sensing. Further studies should clarify which kind of AI-2 structure is recognized by *C. jejuni*.

Future studies could also clarify how the mutation strategy influences gene expression. Variability in the different mutants examined in this study reflects the likely presence of compensatory mutations in other parts of the chromosome. Also polar effects on down- and upstream genes are possible. Further, the influence of mutation strategy on small RNAs cannot be excluded.

One option for further research could be to investigate the expression of the small RNA located downstream of *luxS* in the settings used in this study.
